# Metacognition and diagnostic decision-making: short "blips" of knowledge and the consequences of overconfidence

**DOI:** 10.1186/s41235-026-00717-x

**Published:** 2026-03-13

**Authors:** Seok-sung Hong, Lisa K. Son, Kyungil Kim

**Affiliations:** 1 Social Science Institute, Ajou University, Suwon, Republic of Korea; 2 Barnard College, Columbia University, New York, NY USA; 3 Department of Psychology, Ajou University, Suwon, Republic of Korea

**Keywords:** Overconfidence, Information seeking, Decision-making, Metacognition, Diagnosis

## Abstract

When making decisions under uncertainty, we rely on the information we currently have. Presumably, the more information we have, the better our decisions turn out to be. What, though, drives our motivation to seek more information? The current research investigated people's confidence judgments in one's current information and their relationship to seeking more information when making diagnoses when presented with a sequence of medical symptoms. In Experiment 1, we explored people’s confidence judgments in relation to the accumulation of information they received. In Experiment 2, we tested whether a participant’s confidence levels would guide the decision to seek more information. The results showed that (1) people had the most overconfidence with a short period of exposure to medical knowledge (as opposed to a long period or none at all) and (2) that overconfidence was associated with choosing not to seek more information. In short, a "blip of knowledge" can lead to an illusion of knowing, which, in turn, could lead to a sub-optimal decision when it comes to medical diagnosing.

**Supplementary Information:**

The online version contains supplementary material available at 10.1186/s41235-026-00717-x.

## Significance statement

This study demonstrates that brief exposure to medical information, or a "blip of knowledge," can lead to overconfidence in self-diagnosis, discouraging individuals from seeking additional information. By identifying this cognitive bias and exploring various factors that may influence it, the research provides a foundation for future studies and insights that can help improve decision-making practices and, in the long run, even contribute to better public health outcomes.

**Supplementary Information:**

The online version contains supplementary material available at 10.1186/s41235-026-00717-x.

## Introduction

Imagine waking up one morning with a slight scratch in your throat. You briefly consider calling a doctor, but as a cough develops you reassure yourself that “a cold is going around” and decide that it is probably just a common cold. Most people can relate to such scenarios: When new symptoms appear, we often make an initial judgment about their cause and whether professional help is necessary. In these moments, we act as our own diagnosticians, relying on whatever knowledge we currently have.

Diagnostic decisions are a particularly consequential form of decision-making under uncertainty. One incorrect decision can have serious implications, including, in the extreme case, for morbidity, mortality, and quality of life (Berner & Graber, 2008; Croskerry & Norman, 2008). Although it is impossible to guarantee that every diagnosis is correct, having more relevant information before committing to a diagnosis should, in principle, improve decisions. Yet both clinicians and people frequently stop information search prematurely, sometimes after only a brief exposure to medical information—such as reading a short online article, scanning a search-engine result, or consulting a symptom checker (Bertolazzi et al., 2023; Ceney et al., 2021). The present research examines how such brief exposures to medical knowledge shape people’s confidence in their diagnostic judgments, the accuracy of those judgments, and their willingness to seek additional information.

From a metacognitive perspective, these decisions depend not only on what people know but also on how they monitor and control their knowledge (Koriat, 2012). People must assess how certain they are about a possible diagnosis and then decide whether their current confidence justifies acting on that diagnosis or whether more information should be gathered. A central concern in this process is overconfidence—a miscalibration between subjective confidence and objective accuracy.

### Overconfidence in judgment and decision-making

Overconfidence is one of the most robust and widely documented cognitive biases. People routinely overestimate how well they will perform on tests (Lichtenstein & Fischhoff, 1980), express excessive certainty in their incorrect answers (Fischhoff et al., 1977), and believe that their driving skills (Svenson, 1981) or professional judgments (Wagenaar & Loftus, 1990) are better than they actually are. Following Moore and Healy’s (2008) influential taxonomy, overconfidence can take at least three distinct forms: overestimation (overstating one’s absolute level of performance), overplacement (believing one performs better than others), and overprecision (expressing excessive certainty in the accuracy of one’s beliefs). The present research focuses on overestimation, operationalized as miscalibration between confidence and accuracy in diagnostic judgments. We are interested in whether brief exposure to medical information leads people to become more confident than their actual diagnostic performance justifies. To quantify this form of overconfidence, we adopt a *Bias Index* (BI; Schraw, 2009), defined as the difference between mean confidence and mean accuracy. Positive values indicate that, on average, confidence exceeds accuracy (overconfidence), whereas negative values indicate that confidence falls below accuracy (underconfidence). This measure is well suited to our purposes because it directly captures the calibration between subjective confidence and objective performance, rather than focusing on either variable in isolation.

Overconfidence is not uniform across knowledge levels. The Dunning–Kruger effect is commonly described as low performers providing inflated self-assessments (Kruger & Dunning, 1999; see also Moore & Healy, 2008). Importantly, some work suggests a curvilinear pattern in applied diagnostic contexts, where individuals with some domain knowledge can be more overconfident than those with no knowledge, arguably because minimal knowledge supports plausible—but shallow—explanatory narratives (Sanchez & Dunning, 2018).

### Overconfidence in medical diagnosis and self-diagnosis

Overconfidence is particularly concerning in medical contexts. Clinical research has repeatedly shown that clinicians’ confidence often exceeds their diagnostic accuracy (Miller et al., 2015) and that high confidence can be associated with premature closure—ending diagnostic reasoning too early and failing to consider alternative diagnoses (Berner & Graber, 2008; Cassam, 2017; Croskerry & Norman, 2008). For example, recent work has shown that highly confident physicians make more diagnostic errors and spend less time considering alternative, rarer conditions than their less confident peers (Al-Maghrabi et al., 2024). These data suggest that overconfidence can shorten information search in clinical practice, thereby increasing the risk of error.

People are also increasingly engaged in self-diagnosis, often using online resources, symptom checkers, or large language model-based tools before consulting a physician. While such tools make medical information more accessible, they may also foster a false sense of certainty. Studies of web-based symptom checkers show that their diagnostic and triage accuracy is only moderate, with particular concerns about safety in emergency conditions (Ceney et al., 2021; Kopka et al., 2025). Their use can influence health-care utilization in complex ways, sometimes increasing demand for services and sometimes encouraging self-care without professional consultation (Arellano Carmona et al., 2022).

At the same time, social media and AI-assisted tools are reshaping how people label and understand their health experiences. In youth mental health, for example, many young people arrive at treatment having already self-diagnosed using online information and peer discussions on social media (Armstrong et al., 2025). Recent work on AI-assisted self-diagnosis suggests that large language models can both provide plausible explanations and propagate misleading or incomplete information, potentially amplifying availability biases and medical misinformation (Zada et al., 2025; Zhang et al., 2025). Thus, modern sources of medical information may simultaneously inform and mislead, particularly when individuals base their decisions on limited or poorly integrated knowledge.

### Information seeking and barriers to medical care

Decisions to seek medical treatment are influenced by many factors beyond metacognition, including symptom severity, time and financial constraints, and structural barriers such as lack of health insurance. In the USA, for example, gaps in insurance coverage and high out-of-pocket costs have long prevented many individuals from obtaining needed care (Collins et al., 2012; Doty et al., 2009; Robertson & Collins, 2011), although recent reforms have reduced some of these barriers (Collins et al., 2015). Sociocultural factors also shape help-seeking, particularly in mental health contexts: gender norms, perceived stigma, and coping preferences contribute to differences in willingness to seek psychological treatment (Liddon et al., 2018; Noorwali et al., 2022; Seamark & Gabriel, 2018; Sheu & Sedlacek, 2004).

We do not attempt to model these structural and sociocultural determinants in detail. Instead, we focus on a complementary mechanism that operates “inside the head”—whether overconfidence in one’s own diagnostic judgments discourages further information seeking, even when additional information is readily available at low cost. From a metacognitive standpoint, confidence judgments guide information-seeking behavior—people who feel highly confident may judge that additional information is unnecessary, whereas those who are uncertain may be more likely to seek out more data (Koriat, 2012). If brief exposure to medical information disproportionately increases confidence relative to accuracy, it may lead individuals to stop searching for information prematurely, thereby preventing them from discovering symptoms that point to more serious or less common conditions.

### Information, knowledge, and confidence

Classic work in judgment and decision-making examined how confidence changes as more information becomes available. Fischhoff et al. (1977) introduced the concept of calibration and showed that people’s confidence judgments often deviate systematically from their actual performance. Subsequent research explored how accumulating information interacts with experience and expertise. In a particularly relevant study, Oskamp (1965) asked clinicians to diagnose a single patient across multiple stages, each providing additional clinical information (e.g., more detailed history and test results). With each stage, clinicians’ confidence in their diagnosis increased substantially, whereas diagnostic accuracy improved only modestly. As a result, overconfidence grew as more information was provided. Goldberg (1959) similarly compared clinicians with different levels of experience and found that additional information and experience did not always translate into better calibrated confidence and that simple actuarial models could sometimes outperform human judgment.

These findings suggest that more information does not necessarily yield better calibration; instead, it can sometimes amplify overconfidence. The present research builds on this staged-information paradigm but applies it to people making diagnostic decisions about common illnesses. We ask whether brief, structured exposure to medical information—analogous to quickly reading a symptom list or online article—can produce a short-term boost in confidence that is not matched by a corresponding improvement in diagnostic accuracy. If so, such “blips” of knowledge may be particularly dangerous: They might make people more certain while leaving their underlying understanding shallow.

### The current study

The overarching goal of the present research is to understand how short “blips” of domain-specific information influence overconfidence and information seeking when laypeople make diagnostic judgments under uncertainty. Although our task was administered in a laboratory setting with undergraduate participants, the scenarios were designed to approximate common medical decision contexts encountered by laypersons evaluating their own symptoms (e.g., deciding whether a pattern of cough and fever reflects a cold, influenza, or a more serious condition). Across two experiments, we manipulated the amount of medical knowledge participants received before diagnosing a series of hypothetical patient cases and examined how this manipulation affected diagnostic accuracy, confidence, and the decision to request additional symptom information.

We varied knowledge by assigning participants to one of three conditions: No-Knowledge (NK), Short-Knowledge (SK), and Long-Knowledge (LK). Participants in *No-knowledge* condition received no additional medical information beyond the symptom descriptions presented in the diagnostic task, though they likely possessed some everyday knowledge about common illnesses. Participants in *Short-* and *Long-knowledge* conditions received increasingly extensive, structured information about illnesses and their associated symptoms prior to the task, akin to reading a brief or more detailed online resource. In both experiments, participants diagnosed multiple patient cases using a sequential information paradigm. Each case was presented across three distinct time points, referred to as "stages," with new symptom information revealed cumulatively at each consecutive stage. After each diagnosis, participants rated their confidence, allowing us to compute a *Bias Index* (Schraw, 2009) to assess overconfidence.

Based on prior work on knowledge and overconfidence (Kruger & Dunning, 1999; Moore & Healy, 2008; Oskamp, 1965), we advanced two main hypotheses. First, we predicted a curvilinear relationship between knowledge and overconfidence: brief or partial knowledge (*Short-knowledge*) would produce greater overconfidence than either very limited knowledge (*No-knowledge*) or more extensive knowledge (*Long-knowledge*), even if accuracy improved somewhat with knowledge. In line with common sense and previous research on expertise, we also expected diagnostic accuracy to increase with knowledge, such that participants in *Long-knowledge* condition would be more accurate than those inS*hort-knowledge* and *No-knowledge* conditions. Second, we predicted that higher overconfidence would be associated with reduced information seeking—participants whose confidence exceeded their accuracy to a greater extent would be less likely to request additional symptom information about the patient. By testing these hypotheses in a controlled, yet realistic diagnostic scenario, we aim to clarify how modest increases in medical knowledge can paradoxically increase risk by inflating confidence and discouraging further information search. For transparency, we have retrospectively documented the originally planned two-part study package, a priori hypotheses, and analytic aims in an OSF registration https://osf.io/72udh/overview?vie w_only=e503fda4213d446da9b503d836db3f2d. Because this document was created after data collection and primary analyses, it should be interpreted as a retrospective registration rather than a preregistration.

## Experiment 1

The main question in Experiment 1 was to understand how different amounts of prior medical knowledge and accumulating symptom information jointly shape diagnostic accuracy and confidence. In line with our general hypotheses, we expected diagnostic accuracy to increase with knowledge, particularly in *Long-knowledge* condition, and we predicted that overconfidence (as measured by the *Bias Index*) would be highest in *Short-knowledge* condition relative to both *No-knowledge* and *Long-knowledge* conditions.

### Methods

#### Participants

Eighty-six undergraduate students ($Male=16$, $Female=70$, ${Age}_{M}=20.18 years$, ${Age}_{SD}=3.18 years$) from a women’s liberal arts college located affiliated with a coed university in the USA participated in this experiment (accounting for the large number of women participants). All participants were native English speakers and completed the study in English. A priori power analysis was conducted using G*Power ver. 3.1.9.7 (Faul et al., 2007), treating our 3 (Knowledge: *No-, Short-, Long-knowledge*) × 3 (Stage: 1, 2, 3) mixed design as a standard mixed-design ANOVA for approximation purposes because widely used tools do not provide closed-form power calculations for linear mixed-effects models. This analysis indicated that 69 participants would be sufficient to detect a medium-effect size (d = 0.40) with 95% power (α =.05) for the primary within-between interaction. Our final sample size (N = 86) exceeded this target and should therefore provide at least comparable, if not slightly greater, power for the linear mixed-effects models reported in the Results.

Similarly, based on prior research on clinical versus statistical prediction (Ægisdóttir et al., 2006), it was calculated that 23 participants per group would be required. To ensure sufficient power and account for potential dropouts, we recruited 90 participants. Four participants were excluded due to computer malfunctions. All participants provided informed consent before the experiment and received course credit for their participation. Following previous studies, such as Goldberg (1959), which employed 22 participants, and Oskamp (1965), which included 32 participants, we adopted a similar approach while aligning the sample size with the requirements from our power analysis.

Before conducting the main analyses, we examined participants’ response patterns for evidence of non-differentiated or inattentive responding. Our a priori plan was to exclude any participant who selected the same diagnostic option on every trial or who assigned exactly the same confidence rating on every diagnosis. However, no participants met these exclusion criteria in either experiment, so all collected data were retained for analysis.

#### Design

The experiment used a 3 (Information: Stage 1 vs. Stage 2 vs. Stage 3) within-subjects × 3 (Knowledge: *No-, Short-, Long-knowledge*) between-subjects, mixed design. Traditional repeated-measures ANOVA typically aggregates item-level data, potentially masking variability due to specific disease scenarios (item effects). Furthermore, it treats inter-individual variability primarily as error. By adopting a linear mixed model (LMM), we were able to explicitly model participants and scenarios as random effects. This approach allows for a more robust estimation of the effects of Knowledge and Stage by statistically controlling for the inherent variability in participants' baseline confidence and the varying difficulty levels of different disease scenarios. Participants were randomly assigned to one of the three knowledge conditions before starting the diagnostic task. In *No-knowledge* condition, participants received no additional medical information before the task and relied only on the symptom descriptions presented in the scenarios (plus any everyday knowledge they already had about common illnesses). In *Short-knowledge * condition, participants first studied a script (see Additional file 1: Appendix 2) of common illnesses (e.g., common cold, influenza, measles, tuberculosis) and their typical symptoms for several minutes. In *Long-knowledge* condition, participants studied a more extensive, structured symptom worksheet that listed each target illness along with multiple characteristic symptoms and differentiating features. All materials were provided in a written form, and participants were told that they could use this information to make better diagnoses during the task. In all conditions, they diagnosed the same set of hypothetical patient cases presented across three stages of symptom information. The main dependent variables were confidence and accuracy (of each diagnosis).

#### Stimuli and procedure

The diagnostic task was constructed in several steps. First, we identified sets of illnesses that share similar initial symptoms at Stage 1 (e.g., fever, cough, sore throat), so that the first stage would be relatively ambiguous among the candidate diagnoses. Based on these illness sets, we created 12 multiple-choice patient scenarios in which four illnesses served as response options on each trial. For each vignette, the Stage 1 symptom set was designed so that all four illnesses could plausibly present with those initial symptoms, but it always included at least one symptom that is more typical of the target illness than of the alternatives. Additionally, more differentiating symptoms were then introduced at later stages to gradually increase diagnosability. All scenarios were reviewed by three physicians, who evaluated the clinical plausibility of the symptom combinations and the correctness of the designated target diagnosis. Items for which there was disagreement or concern were revised or discarded. We subsequently conducted a pilot study using the 12 candidate scenarios and retained for the main experiments only those cases whose overall accuracy fell between 40 and 60%. This procedure ensured that the final set of cases avoided items that were trivially easy or impossibly difficult, while preserving realistic confusability between illnesses. We did not attempt to equate the illnesses in terms of their exact base-rate prevalence in the population; instead, our goal was to use illnesses with overlapping early symptoms that non-experts could plausibly confuse and to calibrate item difficulty empirically via the pilot study.

**Fig. 1:**
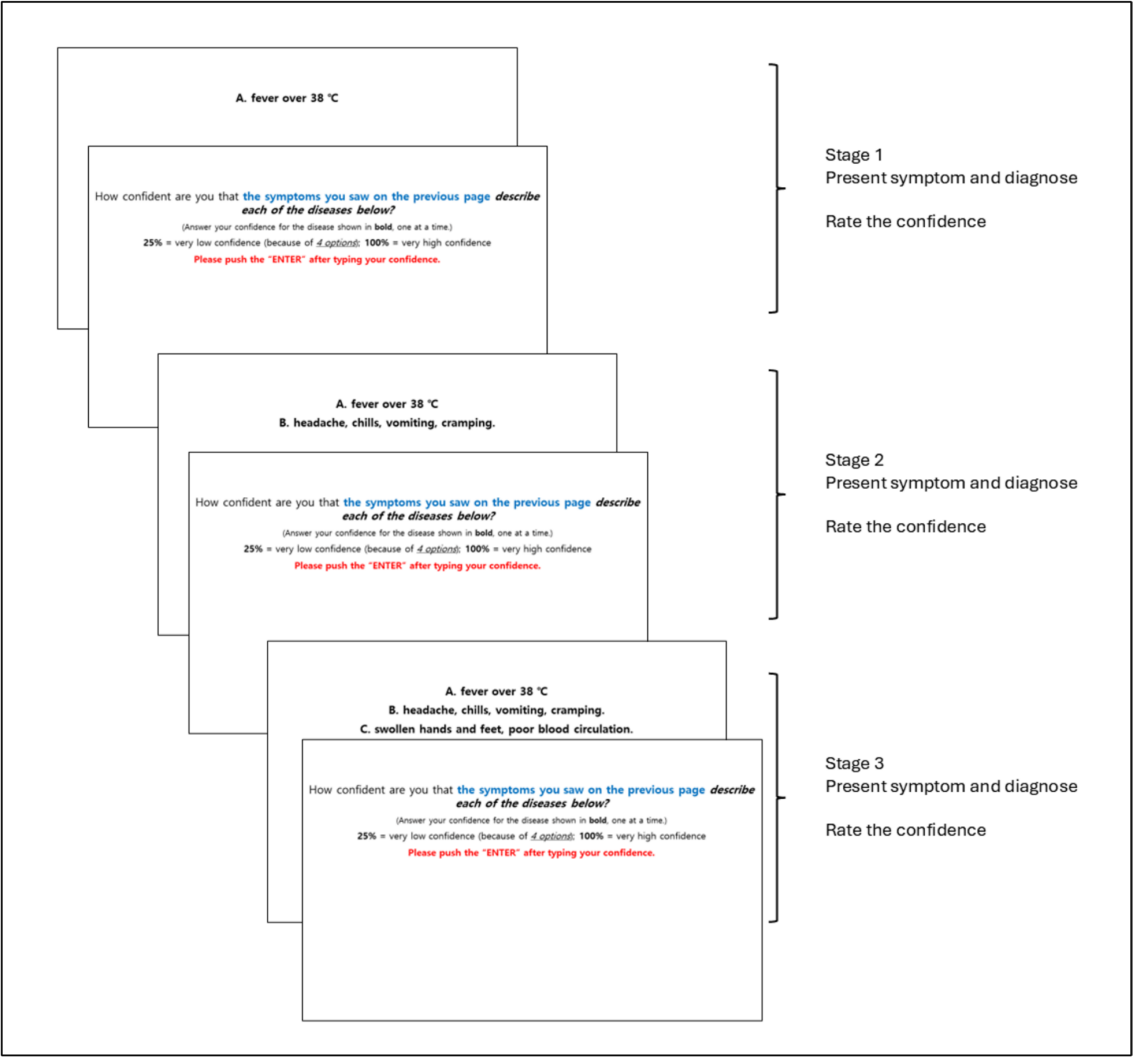
The detailed procedure in Experiment 1

The stimuli were made of a group of medical “symptoms” for a list of 6 diseases (e.g., measles, meningitis, mumps; See the full list of diseases and their symptoms in Additional file 1: Appendix A). For each disease, one new symptom (e.g., fever over 38℃ headaches, chills, vomiting, cramping and swollen hands and feet, poor blood circulation, for Meningitis) was presented for each of 3 "Information" stages (see Fig. 1). For each target disease, symptoms were presented sequentially across three “information” stages (see Fig. 1). At Stage 1, participants saw an initial symptom (e.g., “fever over 38 °C”), selected one of four diagnostic options, and then rated their confidence in this diagnosis on a scale from 25% (low confidence) to 100% (high confidence). They also provided confidence ratings for the three non-selected options. At Stage 2, an additional symptom for the same underlying disease was displayed (e.g., “headache, chills, vomiting, cramping”), followed by another diagnosis and confidence ratings. At Stage 3, a third symptom was added (e.g., “swollen hands and feet, poor blood circulation”), and participants again made a diagnosis and confidence judgments. In this way, participants repeatedly evaluated the same case as new information was added at each stage, closely paralleling Oskamp’s (1965) staged case design in which clinicians reassessed a single patient as additional clinical details became available.

To test knowledge, or expertise, as in the Goldberg (1959) study, we divided participants into three conditions. This manipulation was also used to un-confound "information" (Oskamp, 1965) versus "knowledge" (Goldberg, 1959). In *No-knowledge* condition, participants simply performed the task as described above. In *Short-knowledge* condition, participants were provided with a sheet that listed a series of diseases and their symptoms for 5 min, just prior to starting the experiment. And in *Long-knowledge* condition, participants were provided with that disease sheet for the entire duration of the study, so that they could refer to it when they wanted (see Additional file 1: Appendix B). While this final condition differed from the former two conditions in that the terms were available once the experimental stimuli were presented, we welcomed the fact that it would also be an indication of "seeking more information" even while making online decisions. In other words, participants in *Long-knowledge* condition were not forced by experimenter to refer to the sheet at any time. Indeed, one way to check this would be to look at diagnosis accuracy scores across the conditions at each stage of the experiment.

#### Measures

For each trial and stage, diagnostic accuracy was coded as 1 (correct diagnosis) or 0 (incorrect). Confidence ratings were recorded on a 25–100 scale for the chosen diagnosis. For each participant, we computed mean accuracy and mean confidence separately for each stage and knowledge condition.

To quantify overconfidence, we calculated a *Bias Index* (*BI*) by subtracting accuracy (expressed as a percentage) from confidence. The formula (1) for the *Bias Index*is shown below:

1 $${\mathrm{Bias}}\;{\mathrm{Index}}\left( {{\mathrm{BI}}} \right) = \frac{1}{N}\mathop \sum \limits_{i = 1}^{N} \left( {c_{i} - p_{i} } \right)$$

In this equation, $c_{i}$ represents the confidence rating of participant $i$ (ranging from.25 to 1.00), and $p_{i}$ indicates the performance score (actual accuracy) of participant $i$'s diagnosis (scored as 0 or 1). The *BI* thus reflects the difference between perceived and actual performance, averaged across all trials for each participant. Bias assesses the level to which an individual is over- or under-confident as compared to actual performance, in this case, diagnostic accuracy. While the *Bias Index*is a widely used indicator of overconfidence, it is important to note that this metric can be influenced by both confidence judgments and actual performance. McIntosh et al. (2019) have cautioned against attributing all variation in the *Bias Index*to metacognitive miscalibration without analyzing confidence and accuracy separately. Therefore, while our results show elevated *Bias Index*in *Short-knowledge* condition, further research should disentangle whether this reflects true metacognitive overconfidence or performance-related effects.

In addition to the main task measures, participants provided a self-report rating of their perceived medical knowledge. Specifically, at the end of the experiment they were asked, “How confident would you say you are when it comes to your medical knowledge?” and responded on a 0–100 scale (0 = very low confidence, 100 = very high confidence). This item was used as a simple baseline measure and manipulation check to verify that random assignment to *No-, Short-, *and *Long-knowledge* conditions did not create large pre-existing differences in perceived medical knowledge $\left( {F\left( {2,85} \right) = 2.66,p = .08,\;\eta^{2} = .06} \right)$.

### Results

As a reminder and in line with Goldberg’s (1959) classic finding that additional experience does not necessarily yield better calibrated judgment, we expected diagnostic accuracy to increase with knowledge, particularly in *Long-knowledge* condition, while overconfidence (*Bias Index*) would be greatest in *Short-knowledge* condition. With regard to accumulating symptom information across stages, we anticipated that accuracy would improve only modestly, whereas confidence would increase more strongly, replicating the pattern observed by Oskamp (1965).

#### Accuracy

**Fig. 2:**
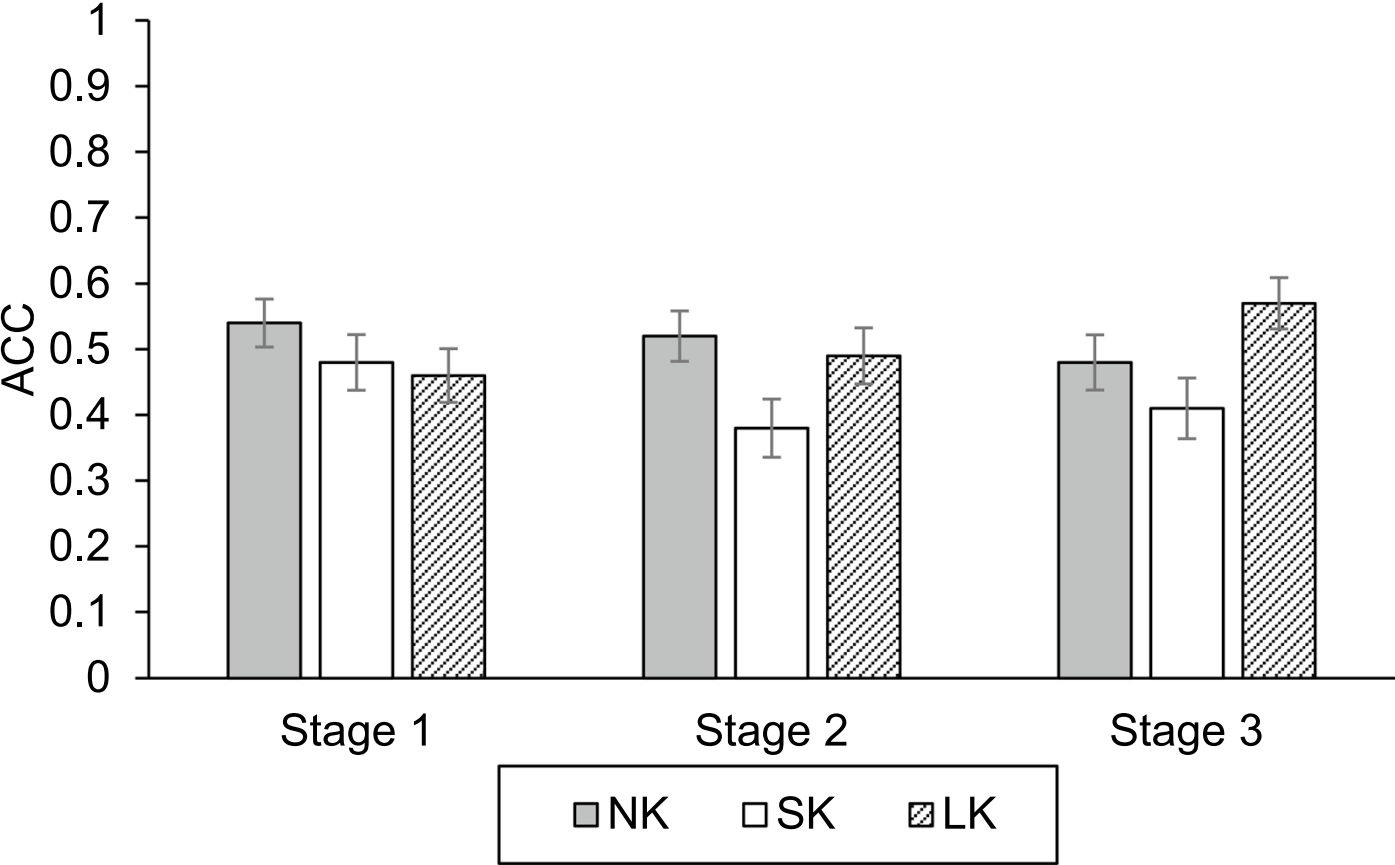
Accuracy across (Information) Stage and (Knowledge) Condition in Experiment 1

The mean accuracies, for each (information) stage and (knowledge) condition, are presented in Fig. 2. We analyzed diagnostic accuracy using a linear mixed model (LMM) to account for random effects associated with participants and specific disease scenarios. The Type III tests of fixed effects revealed a significant main effect of Knowledge, $F\left(\mathrm{2,248.52}\right)=4.51,p<.05$. However, neither the main effect of Stage, $F\left(2, 163.67\right)=0.37,p=.70$, nor the Knowledge × Stage interaction, $F\left(4, 163.67\right)=1.87,p=.12$, was statistically significant.To interpret the main effect of Knowledge, we conducted Bonferroni-corrected pairwise comparisons. The results indicated that *Short-knowledge* condition showed significantly lower accuracy compared to both *No-* (${M diff}_{SK-NK}=-.09, SD=.03, p<.05$) and *Long-knowledge* condition (${M diff}_{SK-LK}=-.09, SD=.03, p<.05$). No significant difference in accuracy was found between *No- and Long-knowledge* conditions ($p=1.00$). Collectively, these findings demonstrate that the reduction in accuracy observed in S*hort-knowledge* condition is a stable effect that persists across information stages. This pattern suggests that a brief exposure to partial knowledge may create a "knowledge blip" that suppresses diagnostic performance relative to both the naive state (*No-knowledge*) and the more informed state (*Long-knowledge*), regardless of the amount of symptom information provided.

#### Bias Index (BI)

Our critical investigation was to see how confidence judgments played out, as a function of information (stages) and/or knowledge (conditions), and in relation to diagnostic accuracy (which, as shown above, was not stable across all three stages or conditions). First, as a check of our confidence ratings, we would expect that the confidence rating would be highest—or tied for highest—for the diagnosis selected (of the 4 options). Indeed, this was the case for our data. We also note that there were more than an insignificant number of times when the selected diagnosis might not have been the sole top choice, in that it shared the top confidence rating with one other (non-selected) diagnosis. This confidence tie occurred on 34.4%, 24.6%, and 19.9%, of the trials at stage 1, 2, and 3, respectively. Such simple data show us that with more information, the "winning" diagnosis seems to emerge, in terms of confidence.

Turning to our main interest, to understand our confidence data regarding error, and to also be able to compare the findings to those of Oskamp (1965) and Goldberg (1959), we looked at the confidence rating people gave for the specific disease that they selected at each stage. We next examined overconfidence using the *Bias Index*, defined as the difference between mean confidence and mean accuracy (see Measures). Table 1 shows the mean *Bias Index* scores by stage and knowledge condition.

**Table 1:** Descriptive statistics for *Bias Index* in Experiment 1

	NK (N = 30)		SK (N = 27)		LK (N = 29)		Total (N = 86)	
	*M*	*SD*	*M*	*SD*	*M*	*SD*	*M*	*SD*
Stage 1	.12	.21	.26	.27	.06	.24	.15	.25
Stage 2	.14	.22	.36	.26	.08	.28	.19	.28
Stage 3	.23	.28	.37	.24	.03	.21	.21	.28

The *Bias Index* for each condition across stages is presented in Table 1. To address the potential influence of individual differences and variability across specific disease scenarios, we conducted a LMM analysis. The model included Knowledge (*No-, Short-, Long-knowledge*) and Stage (1, 2, 3) as fixed effects, while Participant and Item (scenario) were entered as random effects (random intercepts) to control for subject-specific and item-specific variability. The Type III tests of fixed effects revealed a significant main effect of Knowledge, $F(2, 248.28)=25.16$, $p<.001$. However, neither the main effect of Stage, $F(2, 166.73)=1.42$, $p=.25$, nor the Knowledge × Stage interaction, $F(4, 166.73)=1.07$, $p=.37$, was significant. These results indicate that the level of prior knowledge significantly influenced overconfidence (*Bias Index*), regardless of the information stage. To further elucidate the differences between knowledge conditions, we performed Bonferroni-corrected pairwise comparisons based on the estimated marginal means. The results strongly supported the "blip of knowledge" hypothesis. Specifically, participants in *Short-knowledge* condition exhibited a significantly higher *Bias Index* compared to those in *No-* (${M diff}_{SK-NK}=.16$, $SD=.04$, $p<.001$) and *Long-knowledge* condition (${M diff}_{SK-LK}=.27$, $SD=.04$, $p<.001$). Additionally, the *Bias Index* in *No-knowledge* condition was significantly higher than in *Long-knowledge* condition (${M diff}_{NK-SK}=.11$, $SD=.04$, $p<.05$). In summary, even after controlling for random effects associated with participants and items, *Short-knowledge* condition consistently demonstrated the highest level of overconfidence, significantly surpassing both the novice (*No-knowledge*) and expert-like (*Long-knowledge*) conditions. Overall, the data point to an unexpected finding: Confidence, or overconfidence, does not appear to rise linearly.

Numerically, it seemed as though Oskamp's data had been replicated, at least in two of our groups, *No-* and *Long-knowledge* groups. There was an increasing pattern in the *Bias Index* with stage. However, we calculated simple comparisons and found no significant differences, likely due to the high variability.

### Discussion

The most interesting finding in our data was a robust nonlinear relation between knowledge and confidence, validated by our linear mixed model (LMM) analysis. Even after controlling for individual differences and item-specific variability, the "*Short-knowledge*" condition emerged as a distinct outlier, providing a perspective more nuanced than Goldberg’s (1959) classic finding. Goldberg observed that as knowledge increases, overconfidence tends to decrease. Indeed, our LMM results partially support this view: *No-knowledge* group exhibited significantly higher overconfidence than *Long-knowledge* group, suggesting that acquiring substantial expertise does improve calibration. However, the pattern drastically changes when considering partial knowledge. The most striking finding was that participants provided with a brief "blip" of knowledge (*Short-knowledge*) were the most biased, significantly surpassing both the naive (*No-knowledge*) and expert-like (*Long-knowledge*) groups in overconfidence. This implies that while deep knowledge reduces bias, a shallow accumulation of information can paradoxically inflate it—creating an "illusion of knowing" that exceeds even the baseline overconfidence of a novice. Given this robust result, a new concern arises. With the understanding that our metacognitive judgments regulate subsequent control strategies, the possibility of non-optimal strategies becomes a serious issue. That is, if a brief exposure to information leads to peak overconfidence—especially when diagnoses are incorrect—are individuals at greater risk of prematurely ceasing to seek out more information? This was the critical question we addressed in Experiment 2.

## Experiment 2

The main question in Experiment 2 was to see how overconfidence would affect people's decision to seek more information. The results from Experiment 1 suggest that participants were overconfident, particularly those in the group who were exposed to a short period of knowledge. Given these results, we hypothesized that participants in this group would be the least likely to seek more information. In other words, there would be no gap in knowledge to fill.

### Methods

#### Participants

The same 86 undergraduate students who participated in Experiment 1 also took part in Experiment 2. Each participant completed 12 cases in total—6 in Experiment 1 and 6 in experiment 2—but never saw the same case twice. For each participant, 6 of the 12 disease scenarios were randomly assigned to Experiment 1, and the remaining 6 (the ones not used in Experiment 1) were completed in Experiment 2. This random 6–6 split was done independently for each participant. As in Experiment 1, participants signed consent forms prior to starting the experiment and received course credit for their participation. Participants remained in their originally assigned knowledge condition throughout both experiments, and Experiment 2 was administered after Experiment 1 within the same session. No participant saw the same case twice, minimizing item-specific memory effects.

#### Stimuli and procedure

The stimuli and scenarios structure in Experiment 2 were identical to those used in Experiment 1, except with one key addition. Participants were asked about “getting more information or not." At Stage 1, participants saw an initial set of symptoms for a hypothetical patient (e.g., “fever over 38 °C”), selected one of four diagnostic options, and rated their confidence in this diagnosis on a 25–100 scale. Immediately afterward, they were asked whether they wanted more information about this patient (“Would you like more information about this patient?”; Yes/No). If they chose “Yes”, they proceeded to Stage 2, received additional symptoms for the same patient (e.g., “headache, chills, vomiting”), and again made a diagnosis and rated their confidence. They were then asked a second time whether they wanted more information. If they again chose “Yes”, they proceeded to Stage 3, saw a third set of symptoms for the same patient, and repeated the diagnosis and confidence rating.

If at any stage (Stage 1 or Stage 2) they chose “No”, the trial ended for that patient, and they did not see the later-stage symptoms for that case. Thus, in contrast to Experiment 1, where all participants saw all three stages for every case, in Experiment 2 exposure to later-stage symptoms depended on participants’ own information-seeking decisions.

### Results

In this experiment, if participants did not want to get more information after diagnosing stage 1, their diagnostic answers and rating confidence in stage 2 and 3 were recorded as the same as what they had selected in stage 1.

#### Accuracy

**Fig. 3:**
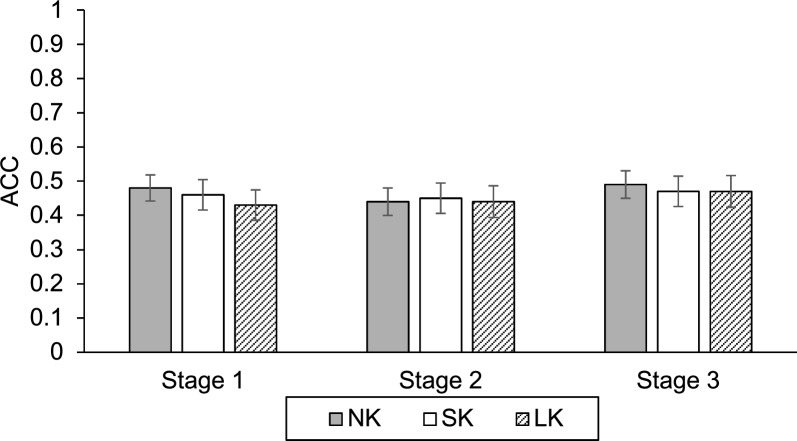
Accuracy by Stage and Condition in Experiment 2. Error bars represent ± 1 standard error of the mean (SEM)

The mean accuracy, for each (information) stage and (knowledge) condition, is presented in Fig. 3. To examine diagnostic accuracy in Experiment 2, we applied the same LMM used in Experiment 1, controlling for participant and item variability. The Type III tests of fixed effects indicated no significant main effects or interactions. Specifically, there was no significant main effect of Knowledge, $F\left(\mathrm{2,248.87}\right)=.32$, $p=.73$, nor a main effect of Stage, $F\left(2, 165.82\right).28$, $p=.76$. The Knowledge × Stage interaction was also not significant, $F\left(\mathrm{4,165.82}\right)=.12$, $p=.98$. Pairwise comparisons further confirmed that there were no statistical differences in accuracy between any of the knowledge conditions (all $p=1.00$).

#### Bias Index (BI)

Thus, more than accuracy, confidence was our key interest. As we did in Experiment 1, we first checked people's confidence ratings, expecting that the confidence ratings would be highest—or tied for highest—for the diagnosis selected (of the 4 options). The data were replicated—each of the selected diseases turned out to have the highest confidence rating. Furthermore, ties decreased across stage: Ties occurred on 35.4%, 25.2%, and 17.6%, of the trials at stage 1, 2, and 3, respectively.

The *Bias Index* for each condition across stages is presented in Table 2.

**Table 2:** Descriptive statistics for *Bias Index* in Experiment 2

	NK (N = 30)		SK (N = 27)		LK (N = 29)		Total (N = 86)	
	*M*	*SD*	*M*	*SD*	*M*	*SD*	*M*	*SD*
Stage 1	.16	.26	.28	.28	.12	.25	.18	.27
Stage 2	.22	.26	.32	.26	.13	.26	.22	.27
Stage 3	.18	.26	.33	.24	.12	.25	.21	.26

We analyzed the *Bias Index* using a LMM to account for random effects associated with participants and specific disease scenarios. The Type III tests of fixed effects revealed a significant main effect of Knowledge, $F\left(\mathrm{2,248.55}\right)=11.30,p<.001$. However, the main effect of Stage was not significant, $F\left(\mathrm{2,169.37}\right)=.51,p=.60$, and the Knowledge × Stage interaction was also non-significant, $F\left(\mathrm{4,169.37}\right)=.17,p=.96$. To interpret the main effect of Knowledge, we performed Bonferroni-corrected pairwise comparisons. The results replicated the "knowledge blip" pattern observed in Experiment 1. Specifically, participants in *Short-knowledge* condition exhibited a significantly higher *Bias Index* compared to both *No-* (${M diff}_{SK-NK}=.12, SD=.04, p<.01$) and *Long-knowledg*e condition (${M diff}_{SK-LK}=.19, SD=.04, p<.001$). There was no statistically significant difference in bias between *No-* and *Long-knowledge* conditions ($p=.31$). These findings confirm that the elevated overconfidence in *Short-knowledge* condition is a robust phenomenon that persists across information stages when controlling for participant and item variability. The results suggest that a brief exposure to knowledge significantly inflates confidence relative to accuracy, regardless of the amount of symptom information provided.

#### Seeking more information

**Fig. 4:**
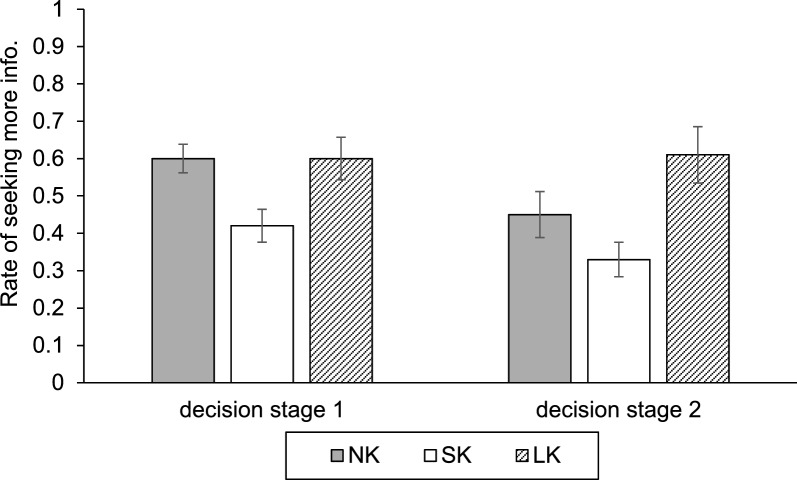
The rate of “seeking more information” by Stage and Condition in Experiment 2. Error bars represent ± 1 standard error of the mean (SEM)

Our primary question in Experiment 2 was to understand whether there would be a relationship between confidence and "seeking more information." Fig. 4 (also in Table 3) presents the rate of seeking more information first by condition, at stage 1 (for receiving information in stage 2) and at stages 2 (for receiving information in stage 3). To investigate participants’ willingness to seek additional information, we analyzed the rate of information seeking using a LMM to control for random effects associated with participants and disease scenarios. The Type III tests of fixed effects revealed a significant main effect of Knowledge, $F\left(\mathrm{2,144.51}\right)=7.04,p<.001$. However, the main effect of Decision Stage was not significant, $F\left(\mathrm{2,144}.50\right)=.03,p=.87$, nor was the Knowledge × Decision Stage interaction, $F\left(\mathrm{2,144.51}\right)=1.43,p=.24$. To clarify the main effect of Knowledge, we performed Bonferroni-corrected pairwise comparisons. The results indicated that participants in *Short-knowledge* condition were significantly less likely to seek more information compared to those in *No-* (${M diff}_{SK-NK}=-.15, SD=.06, p<.05$) and *Long-knowledge* condition (${M diff}_{SK-LK}=-.22, SD=.06, p<.001$). There was no significant difference in information seeking between *No-* and *Long-knowledge* conditions ($, p=.70$). In sum, participants in *Short-knowledge* condition—who exhibited the highest overconfidence (*Bias Index*)—consistently demonstrated the lowest willingness to seek additional diagnostic information. This tendency to prematurely stop information seeking was robust across decision stages, distinguishing *Short-knowledge* group from both the novice (*No-knowledge*) and expert-like (*Long-knowledge*) groups.

**Table 3:** Descriptive statistics for the rate of “getting more information” in Experiment 2

	NK (N = 27)		SK (N = 23)		LK (N = 24)		Total (N = 74)	
	*M*	*SD*	*M*	*SD*	*M*	*SD*	*M*	*SD*
Decision stage 1	.60	.20	.42	.21	.60	.28	.54	.24
Decision stage 2	.45	.32	.33	.22	.61	.37	.46	.33

## General discussion

The results of our study suggest that even a brief “blip” of domain-specific information can foster substantial overconfidence and that this overconfidence, in turn, shapes subsequent decision-making. Before discussing these core findings, it is worth noting a nuance regarding diagnostic accuracy. In Experiment 2, we found no significant differences in accuracy across knowledge conditions. This suggests that the knowledge script provided to participants may not have functioned as a direct “answer key.” Even with access to the symptom sheet, considerable ambiguity remained in the symptom-to-diagnosis mappings (e.g., a “cough” could indicate influenza, a cold, or COVID-19). This inherent ambiguity implies that accuracy alone may not fully capture the impact of partial knowledge; rather, the critical role of such knowledge appears to be in how it shapes participants’ confidence—and consequently, their willingness to engage in further information seeking.

A critical insight emerges from the comparison between the two experiments. In Experiment 1, where participants were forced to process all information stages, *Short-knowledge* condition exhibited a significant "suppression effect," performing worse in accuracy than both *No-* and *Long-knowledge* conditions. However, in Experiment 2, where participants had the autonomy to stop information seeking, this performance gap disappeared. The absence of significant accuracy differences in Experiment 2 suggests that when the decision process allows for voluntary cessation, the specific performance disadvantage of the "knowledge blip" observed in the forced-exposure context is attenuated. This highlights that the danger of partial knowledge lies not necessarily in the inability to diagnose, but in the premature closure of the diagnostic process driven by inflated confidence. Regarding this confidence, our linear mixed model (LMM) analyses across both experiments confirmed a robust pattern: participants with a "blip" of knowledge (*Short-knowledge*) showed significantly higher confidence (*Bias Index*) than both novices (*No-knowledge*) and those with extensive knowledge (*Long-knowledge*), even after controlling for individual and item-specific variability. This pattern held regardless of diagnostic accuracy.

Critically, we found that this overconfidence had downstream behavioral consequences. Consistently, participants in the *Short-knowledge* condition—those showing the largest bias—were the least likely to request additional information when given the opportunity. These findings align well with Metcalfe and Finn’s (2008) proposal that metacognitive judgments regulate study choices. Our results indicate that inaccurate monitoring (inflated confidence from a "blip") distorts control behavior (reduced information seeking), consistent with classic metacognitive control models.

Theoretically, these results refine the expectations set by prior work. Following Oskamp (1965), we expected that increasing information across stages would primarily increase confidence rather than accuracy. Following Goldberg (1959), we anticipated that greater, more structured knowledge might dampen confidence or improve calibration. In line with Oskamp, accuracy increased only modestly whereas confidence tended to rise as more symptom information was presented, and this pattern was most evident in *No-* and *Short-knowledge* conditions. In line with Goldberg, confidence was lower in *Long-knowledge* condition than in *Short-knowledge* condition. However, when *No-knowledge* group is considered alongside *Short-* and *Long-knowledge*, the overall relationship between knowledge and confidence appears nonlinear: a short “blip” of knowledge (*Short-knowledge*) produced the greatest overconfidence, rather than a simple monotonic increase or decrease with knowledge.

More broadly, these findings speak to the kinds of “knowledge” that are increasingly prevalent in an internet-driven world. People are constantly exposed to small fragments of information—often only a few minutes or seconds at a time—about complex domains such as medicine. If such brief exposures confer a sense of unwarranted confidence without commensurate gains in accuracy, subsequent decisions may be altered for the worse. In particular, if a short blip of medical information encountered online is treated as equivalent to professional advice, the consequences for diagnosis and treatment decisions can be serious.

A further limitation of the present work concerns the cultural context of our sample. Both experiments were conducted with undergraduate students an institution in US, and all participants completed the tasks in English. Attitudes toward illnesses such as influenza or COVID-19, as well as norms surrounding prevention, vaccination, and health-care seeking, may differ across cultures. These cultural differences could in turn influence how people interpret symptom information, how confident they feel about their diagnoses, and how willing they are to request additional information. Accordingly, the present findings should be interpreted with caution when generalizing beyond US undergraduate populations. An important direction for future research will be to examine whether similar patterns of diagnostic overconfidence and information seeking emerge in more diverse and cross-cultural samples.

Another limitation is that, although we obtained a simple self-report measure of participants’ overall medical knowledge (0–100), we did not collect detailed information about their prior exposure to or experience with each of the target illnesses. For instance, we do not know whether some participants had previously been diagnosed with influenza, measles, or other relevant conditions, or whether close others had experienced these illnesses. Such personal experience could plausibly influence both diagnostic accuracy and confidence, as well as willingness to seek additional information. Future studies should therefore include more fine-grained measures of illness history and exposure (e.g., self-reported prior diagnoses, family history, vaccination status) to clarify how prior experience shapes overconfidence and information seeking in medical judgment.

A further methodological limitation concerns our lack of direct measures of participants’ engagement with the knowledge materials in *Short-* and *Long-knowledge* conditions. Although we manipulated brief scripts versus extended symptom worksheets and observed reliable differences in confidence and information seeking across conditions, we did not record how much time participants spent studying these materials, how often they consulted the worksheets during the diagnostic task, or how well they actually learned the content. As a result, our interpretation of *No-, Short-,* and *Long-knowledge* as differing levels of implemented knowledge rests on the logic of the manipulation and on participants’ self-reported medical knowledge, rather than on direct process measures of worksheet use. Future research should therefore incorporate more fine-grained indicators of engagement and learning (e.g., study time, access logs, eye-tracking, or post-test quizzes on worksheet content) to more precisely link knowledge acquisition, metacognitive monitoring, and information-seeking behavior.

A related direction concerns preventive health behaviors such as vaccination. In the present studies, we did not collect data on participants’ vaccination history or willingness to be vaccinated for illnesses such as measles, influenza, or tuberculosis, because our primary goal was to isolate how information, knowledge, and experience shape information-seeking behavior in a controlled medical domain. We chose diagnostic scenarios precisely because they allowed us to hold constant a basic level of lay familiarity with common illnesses while systematically manipulating the amount and format of knowledge. Nonetheless, the current paradigm could be readily extended to examine how overconfidence and reduced information seeking relate to attitudes toward vaccination and other preventive decisions. For example, future work could test whether individuals who are less willing to seek additional diagnostic information are also less inclined to accept vaccination recommendations, and how curiosity and shared decision-making processes might mediate this relationship.

Overall, the present studies extend classic work on overconfidence by demonstrating how partial knowledge can simultaneously inflate confidence and suppress information seeking in an applied diagnostic setting. We show that overconfidence is not merely a distortion in metacognitive monitoring; it also has direct implications for control, in this case a premature cessation of information seeking. We hope that these findings will stimulate further research on metacognitive assessments in a range of applied contexts, especially those that combine today’s information technologies with lay medical decision-making. As people are exposed to ever-increasing amounts of fragmented information, understanding how such “blips” of knowledge shape confidence and choice—and how factors such as social media use may further amplify illusions of knowing—will be an important task for future work.

## Supplementary Information

Additional file 1.

## Data Availability

All data (Excel and SPSS files), SPSS analysis syntax, and research materials (instruction text, images, and E-Prime 3.0 files) are available on the Open Science Framework at [data/materials link]. In addition, the originally planned two-part study package, a priori hypotheses, and analytic aims have been documented in a retrospective OSF registration at https://osf.io/72udh/overview?view_only=e50 3fda4213d446da9b503d836db3f2d. Because this registration was created after data collection and primary analyses, it is not a preregistration.
