# Correction: Metacognition and diagnostic decision-making: short "blips" of knowledge and the consequences of overconfidence

**DOI:** 10.1186/s41235-026-00742-w

**Published:** 2026-07-13

**Authors:** Seok-sung Hong, Lisa K. Son, Kyungil Kim

**Affiliations:** 1 Social Science Institute, Ajou University, Suwon, Republic of Korea; 2 Barnard College, Columbia University, New York, NY USA; 3 Department of Psychology, Ajou University, Suwon, Republic of Korea

**Correction to: Cognitive Research: Principles and Implications (2026) 11:22** 10.1186/s41235-026-00717-x

The original article has been updated to amend various minor typographical errors which were mistakenly carried forward by the production team which handled the article.

