# Supplementary material for: Metacognition and diagnostic decision-making: short "blips" of knowledge and the consequences of overconfidence
Source: Cogn Res Princ Implic. 2026 Mar 13;11:22. doi: 10.1186/s41235-026-00717-x (PMC12988131; doi:10.1186/s41235-026-00717-x)

# Appendix A

**Scenario 1.**

fever over 38 ℃

headache, chills, vomiting, cramping.

swollen hands and feet, poor blood circulation.

1. Hand-foot-and-mouth disease
2. Influenza
3. Laryngitis
4. **Meningitis**

**Scenario 2.**

fever, headache, chills, vomiting, muscle pain.

infection inside mouth, toothache.

pain between ears and neck.

1. Laryngitis
2. **Mumps**
3. Brain tumor
4. Ear infection

**Scenario 3.**

stomachache after eating, loss of appetite and/or weight, jaundice.

vomiting, thirst, fatigue, slow recovery from injury, frequent urination.

shock and bleeding in the stomach and/or intestines.

1. Hepatitis
2. Diabetes
3. Gastritis
4. **Pancreatic cancer**

**Scenario 4.**

cough, sputum

pressure on chest, difficulty in breathing

frequently waking from cough, in severe cases, seizures.

1. **Asthma**
2. Tuberculosis
3. Pneumonia
4. Cold

**Scenario 5.**

backache, nausea, vomiting, frequent urination.

sharp pain in your core.

blood in the urine, pain in lower abdomen and genital area.

1. Menopause
2. cervical cancer
3. **Urinary stone**
4. Prostate cancer

**Scenario 6.**

small lump in armpit, nipple retraction.

pain in breasts, nipple discharge.

Swollen, red ulcers on skin.

1. Muscle pain
2. Breast engorgement
3. **Breast cancer**
4. Armpit enlargement lymph node

**Scenario 7.**

Hand/leg cramps, and delays in action.

Frequent urination, low blood pressure when standing up.

stiffness at the joints, numbness, burning sensation over the body.

1. Brain tumor
2. **Parkinson’s disease**
3. Amyotrophic Lateral Sclerosis (ALS)
4. Seizure

**Scenario 8.**

Fever, runny nose.

Cough, vomiting.

Conjunctivitis in the mouth, body rash.

1. **Measles**
2. Hand-foot-and-mouth disease
3. Laryngitis
4. Enteritis

**Scenario 9.**

Sneezing, runny/stuffy nose

Itchy, dry eyes

Loss of sense of smell, ringing in the ears.

1. Cold
2. **Rhinitis**
3. Pollen allergy
4. Influenza

**Scenario 10.**

Decreased motivation, insomnia, decline in attention.

Negative thinking, change in weight, increasing or decreasing appetite.

Continuous feelings of sadness, in severe cases, attempting suicide.

1. Bipolar Disorder
2. Mood Disorder
3. Panic disorder
4. **Depressive disorder**

**Scenario 11.**

Cough, sputum, fever.

Loss of appetite, indigestion, headache.

Hemoptysis, bloody sputum, loss of weight.

1. **Tuberculosis**
2. Lung cancer
3. Vertigo
4. Asthma

**Scenario 12.**

Runny nose, nasal congestion, cough, headache.

Fever, throat pain, muscle pain, sputum.

Feeling drying in throat, conjunctivitis, fatigue.

1. Meningitis
2. Pneumonia
3. **Cold**
4. Hand-foot-and-mouth disease

# Appendix B
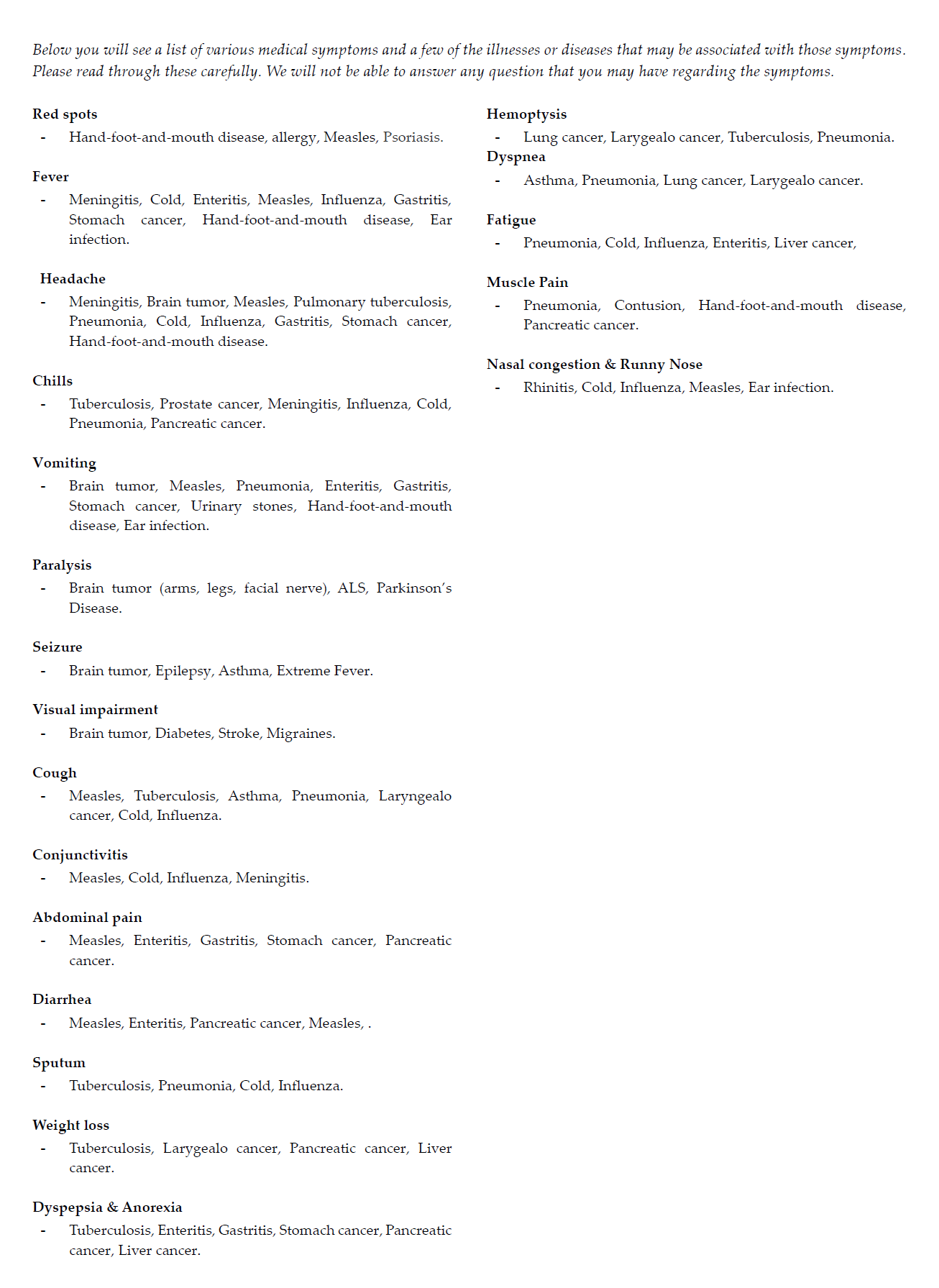

Supplement: Supplementary file 1 — Additional file 1. [file 41235_2026_717_MOESM1_ESM.docx]
